# Supplementary material for: Carbon accumulation in recently deposited peat is reduced by increased nutrient supply
Source: Nat Commun. 2025 May 8;16:4271. doi: 10.1038/s41467-025-59387-w (PMC12062222; doi:10.1038/s41467-025-59387-w)
Supplement: Supplementary file 1 — Supplementary information [file 41467_2025_59387_MOESM1_ESM.pdf]

Nature communications

Supporting Information for

Carbon accumulation in recently deposited peat is reduced by increased nutrient supply

Betty Ehnvall[1,2,*], Joshua L. Ratcliffe[1,3], Carolina Olid[4], Jacob Smeds[1], Kevin Bishop[2], Jonatan Klaminder[1], Chuxian Li[2,5], Carl-Magnus Mörth[6], Mats B. Nilsson[1], Mats G. Öquist[1]

1. Department of Forest Ecology and Management, Swedish University of Agricultural Sciences, Skogsmarksgränd 17, 90183 Umeå, Sweden
2. Department of Aquatic Sciences and Assessment, Swedish University of Agricultural Sciences, 75007, Uppsala, Sweden
3. Unit for Field-Based Forest Research, Swedish University of Agricultural Sciences, 922 91, Vindeln, Sweden
4. UB-Geomodels Research Institute, Departament de Dinàmica de la Terra i l'Oceà, Facultat de Ciències de la Terra, Universitat de Barcelona, 08028 Barcelona, Spain
5. Institute of Geography and Oeschger Center for Climate Change Research, University of Bern, 3012 Bern, Switzerland
6. Department of Geological Sciences, Stockholm University, Svante Arrheniusväg 8, 10691 Stockholm, Sweden
* Author for correspondence: betty.ehnvall@slu.se

A. Background information

Table S1. Sampling point properties

ID	Latitude	Longitude	Elevation (m.a.s.l.)	Surface age (years)[a]	Peat depth (cm)	Ground-water level[b] (cm)	pH
S70	63°51′9″N	20°42′35″E	1.5	150	46	9.1 ± 6.7	4.0 ± 0.2
S10	63°49′9″N	20°34′42″E	5.1	500	140	8.0 ± 4.5	4.1 ± 0.3
S52	63°57′17″N	20°46′15 ″E	12.6	1,200	114	2.2 ± 5.3	4.7 ± 0.2
S18	63°53′8″N	20°43′48″E	14.5	1,400	66	17.5 ± 4.4	3.6 ± 0.1
S26	63°52′5″N	20°30′29″E	29.2	2,700	246	12.1 ± 4.3	3.7 ± 0.2
S65	63°52′58″N	20°38′50″E	34.8	3,150	130	12.8 ± 4.3	3.8 ± 0.2
1106	63°55′10″N	20°26′ 48″E	46.7	4,000	169	17.6 ± 7.6	4.1
1639	63°58′9″N	20°27′ 23″E	53.6	4,500	151	18.2 ± 5.8	4.5

a. Estimated from shore displacement curve[1] and the elevation above sea level.
b. Average relative water table depth June 1-August 31 based on years 2022-2023 for mires S70, S10, S52, S18, S26 and S65, on year 2023 for mires 1106 and 1639. Uncertainty corresponds to standard deviation over each applied time period.

B. OPLS models

Table S2. OPLS model statistics for peat mass (MAR), carbon (CAR) and nitrogen (NAR) accumulation rates for lawns and hummocks separately.

	Sig.[a]	R^2Cum	Q^2Cum	RMSEE	RMSEcv	MBEcv
MAR lawn	S+S	0.99	0.99	-	1.60	1.50
MAR hummock	NS+NS	0.99	0.86	4.01	14.14	26.63
CAR lawn	S+S	0.99	0.99	-	0.05	0.04
CAR hummock	NS+NS	0.99	0.86	1.75	8.18	6.40
NAR lawn	S+S	0.99	0.99	-	0.05	0.04
NAR hummock	NS+NS	0.99	0.87	0.01	0.12	0.10
Prod lawn	NS+NS	0.92	0.25	0.01	0.02	<0.01
Decay lawn	NS+NS	0.99	0.78	<0.01	<0.01	<0.01

a. Significance of predictive (first) and orthogonal (second) component. S corresponds to significant at $Q^2 < 0.01$ and NS to non-significant at $Q^2 < 0.01$.

Table S3. Predictors included in the OPLS models

Predictor	Description	Data source
Age	Mire age (years) by the sampling points calculated from the surface elevation using shore displacement curve.	DEM (Swedish Mapping, Cadastral and Land Registration Authority), shore displacement curve[1]
Al, C, Ca, Fe, K, N, Na, Mg, Mn, P, Si, S, Zn	Elemental concentrations (mg g^{-1}) in surface peat.	Wang et al.[3]
C/N	Carbon-to-nitrogen ratio in surface peat.	50 cm peat core, total carbon and nitrogen mass ratios
Depth	Total peat depth (m) by the sampling points.	Manual measurements[2,3]
GWL	Groundwater level (cm) relative to mire surface for years 2022-2023[a] and 2023[b].	Monitored data using Odyssey® Xtreem Capacitance Water Level Loggers and Levellogger 5 Junior
Area(M)	Total mire area (ha).	The Swedish Property Map (Swedish Mapping, Cadastral and Land Registration Authority)
NDVI	Sampling point mean normalized difference vegetation index (10 x 10 m raster) for July based on years 2017-2023.	Sentinel-2 Level 2A (European Space Agency)
pH	Acidity in surface peat.	Ehnvall et al.[2]; Wang et al.[3]
Area(C)	Total area (ha) of the upslope catchment area that a mire does not share with any upslope mire.	DEM (Swedish Mapping, Cadastral and Land Registration Authority; Ehnvall et al.[2])
Elev(C)	Mean elevation (meters above sea level) of the unique upslope catchment area that a mire does not share with any upslope mire.	DEM (Swedish Mapping, Cadastral and Land Registration Authority; Ehnvall et al.[2])
C-to-M	Catchment-to-mire areal ratio	The Swedish Property Map (Swedish Mapping, Cadastral and Land Registration Authority), DEM (Swedish Mapping, Cadastral and Land Registration Authority; (Ehnvall et al.[2])
Peat(C)	Mean peat depth (cm) in the upslope catchment area that a mire does not share with any upslope mire.	DEM (Swedish Mapping, Cadastral and Land Registration Authority; Ehnvall et al.[2]), Peat depth map[4]
Slope(C)	Mean slope in the unique upslope catchment area that a mire does not share with any upslope mire.	DEM (Swedish Mapping, Cadastral and Land Registration Authority)
SMI(C)	Mean soil moisture in the unique upslope catchment area that a mire does not share with any upslope mire.	DEM (Swedish Mapping, Cadastral and Land Registration Authority), Soil moisture map (Swedish university of Agricultural Sciences)

a. Mires S10, S18, S26, S52, S65, S70
b. Mires 1106, 1639

C. Pb-210 profiles

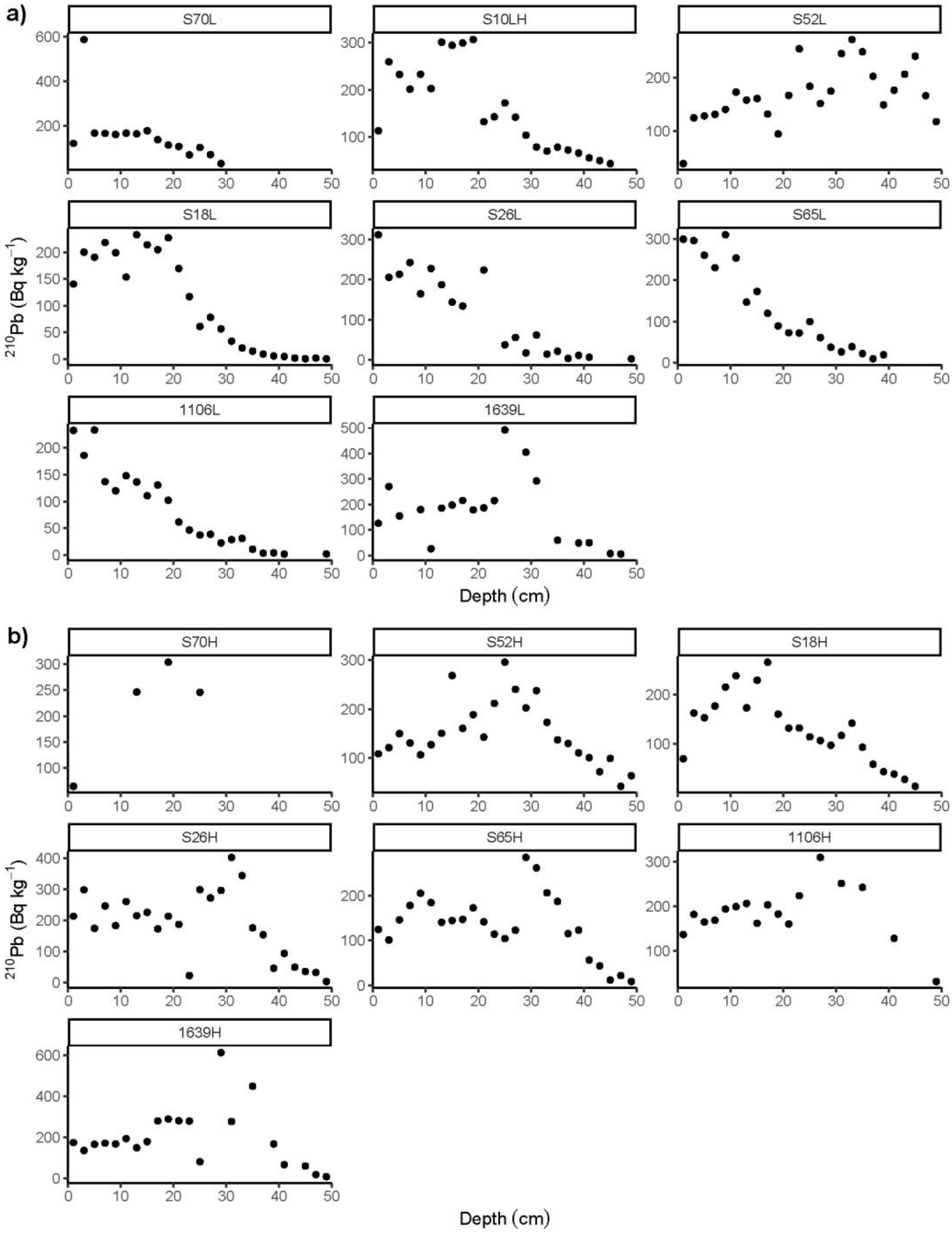

Figure S1. Pb-210 profiles in (a) lawns and (b) hummocks sorted from youngest (S70) to oldest (1639) chronosequence mire.

D. Carbon and nitrogen concentrations in the peat profiles

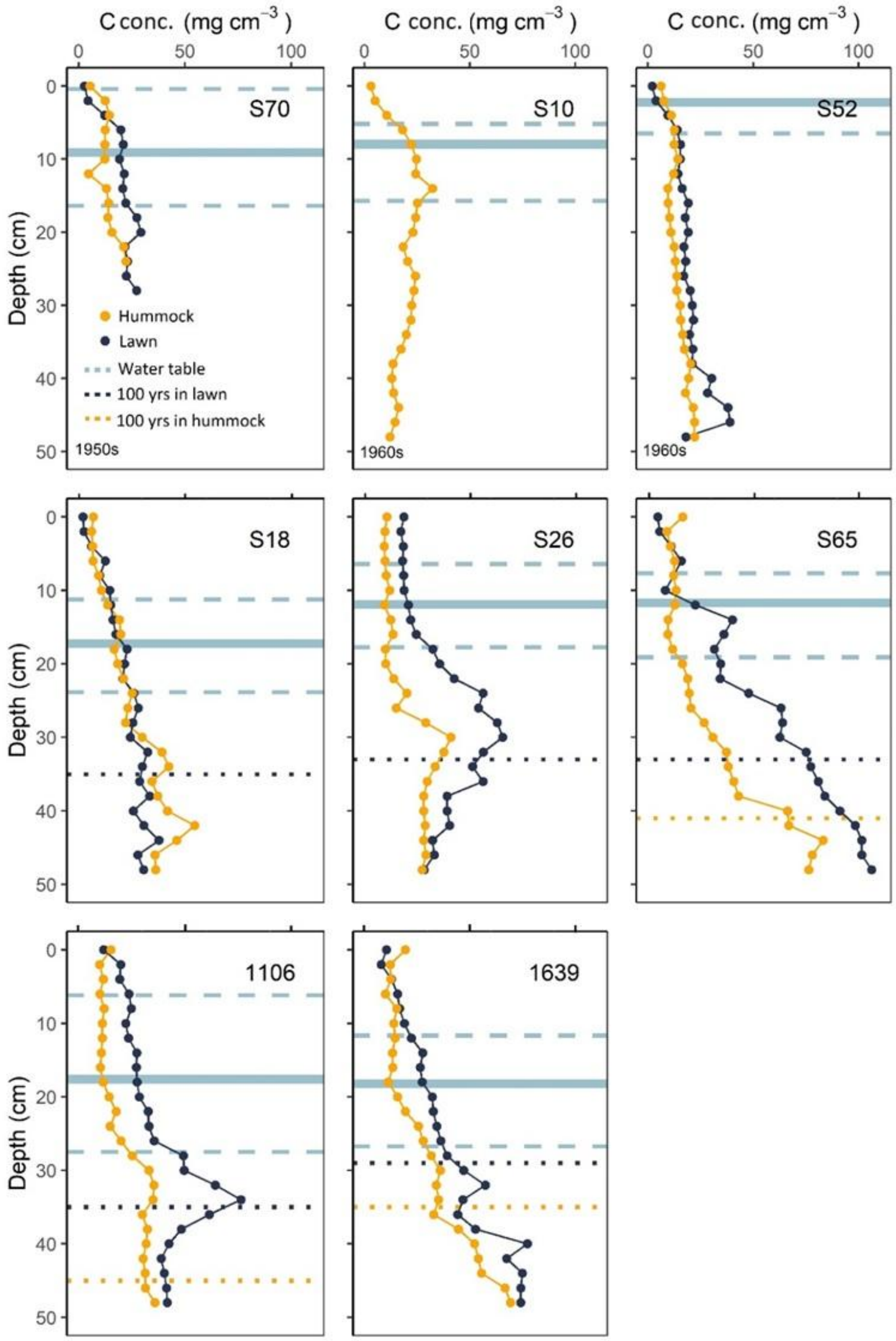

Figure S2. Carbon concentration (mg cm^{-3}) profiles. Light blue lines mark the 10th (dotted), the 50th (i.e. median; solid) and 90th (dotted) percentiles of water table level over the vegetation period (June 1-August 31) over the years 2022-2023 for S70, S10, S52, S18, S26 and S65, and over year 2023 for 1106 and 1639. Dark blue and yellow dotted lines correspond to the estimated peat depth 100 years before sampling (i.e. around the 1920s) based on ^{210}Pb chronologies, for lawns and hummocks respectively. In S70, S10 and S52 background levels of ^{210}Pb were not reached in the sampled cores. For these mires, the oldest registered peat is reported in the bottom left corner of the panel (S70 lawn, S52 hummock, S10 – no microtopography).

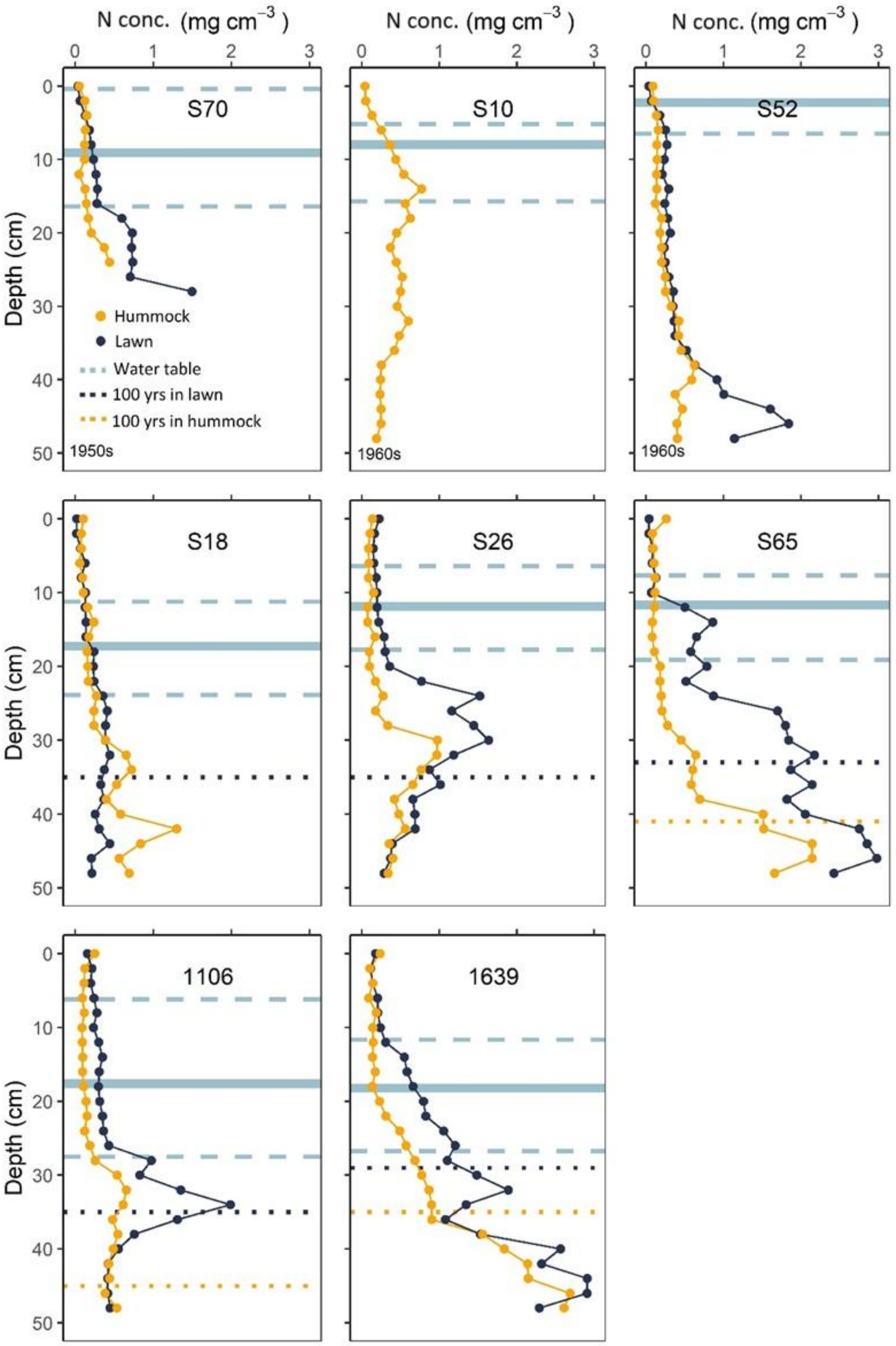

Figure S3. Nitrogen concentration (mg cm^{-3}) profiles. Light blue lines mark the 10th (dotted), the 50th (i.e. median; solid) and 90th (dotted) percentiles of water table level over the vegetation period (June 1-August 31) over the years 2022-2023 for S70, S10, S52, S18, S26 and S65, and over year 2023 for 1106 and 1639. Dark blue and yellow dotted lines correspond to the estimated peat depth 100 years before sampling (i.e. around the 1920s) based on ^{210}Pb chronologies, for lawns and hummocks respectively. In S70, S10 and S52 background levels of ^{210}Pb were not reached in the sampled cores. For these mires, the oldest registered peat is reported in the bottom left corner of the panel (S70 lawn, S52 hummock, S10 – no microtopography).

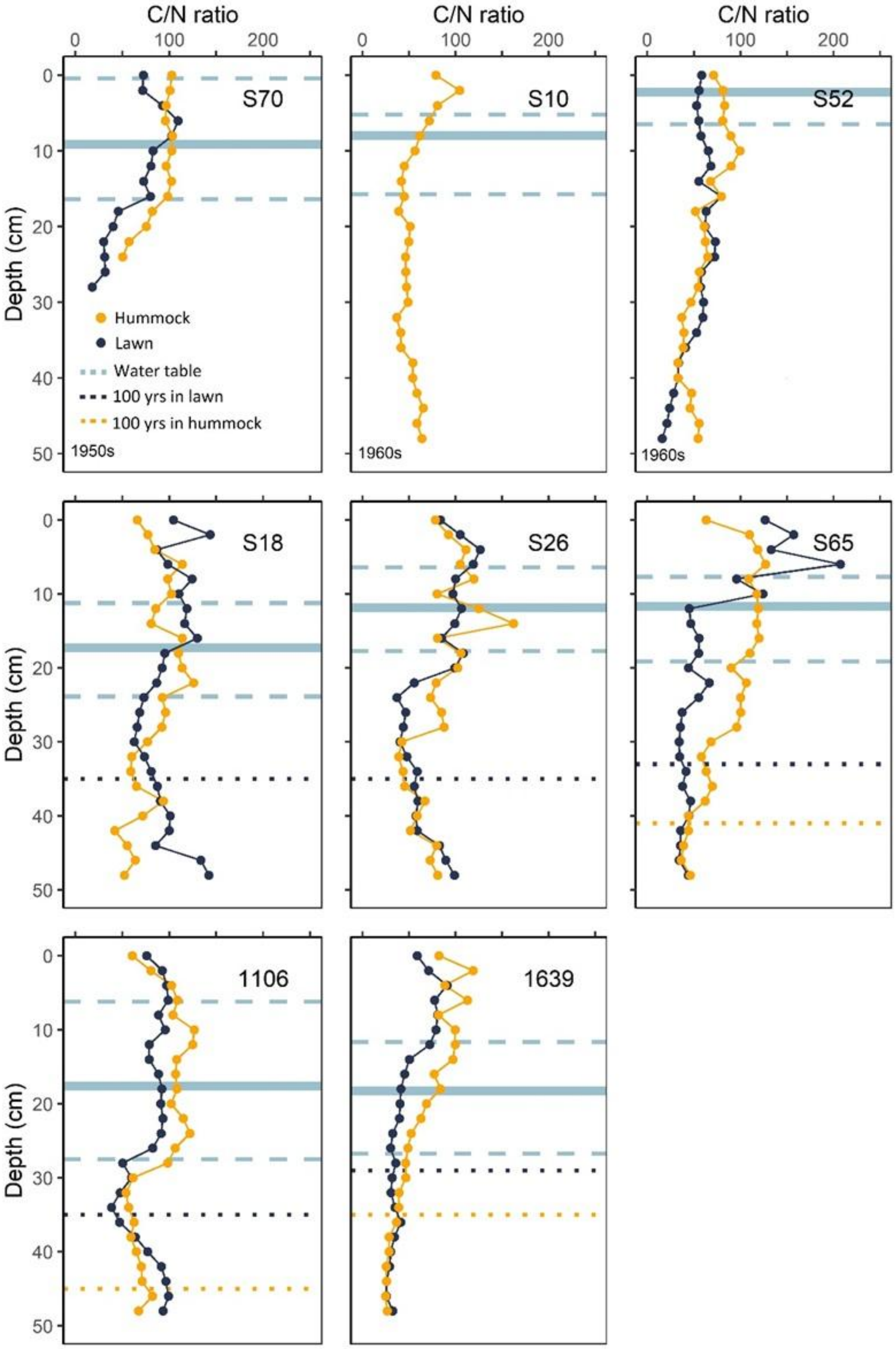

Figure S4. Carbon-to-nitrogen (C/N) profiles. Light blue lines mark the 10th (dotted), the 50th (i.e. median; solid) and 90th (dotted) percentiles of water table level over the vegetation period (June 1-August 31) over the years 2022-2023 for S70, S10, S52, S18, S26 and S65, and over year 2023 for 1106 and 1639. Dark blue and yellow dotted lines correspond to the estimated peat depth 100 years before sampling (i.e. around the 1920s) based on ^{210}Pb chronologies, for lawns and hummocks respectively. In S70, S10 and S52 background levels of ^{210}Pb were not reached in the sampled cores. For these mires, the oldest registered peat is reported in the bottom left corner (S70 lawn, S52 hummock, S10 – no microtopography).

E. Peat productivity and decay rates in hummocks and lawns

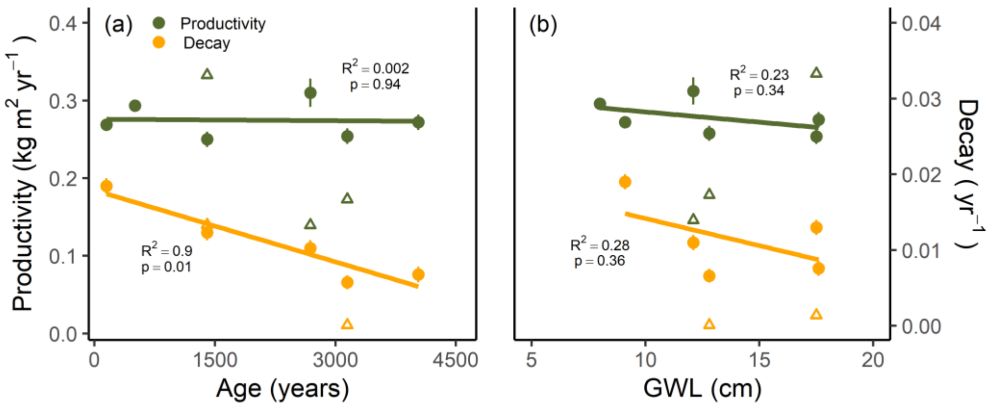

Figure S5. Peat productivity (green) and decay rates (orange) in lawns derived from Clymo's model on peat accumulation relative to mire age (a) and water table level (b). Peat productivity refers to the gross peat formation, i.e. the addition of new peat by the mire surface, while the decay refers to the decay constant. Triangles correspond to peat productivity (green) and decay (orange) in hummocks. These do not contribute to the regression.

References

1. Renberg, I. & Segerström, U. The initial points on a shoreline displacement curve for southern Västerbotten, dated by varve-counts of lake sediments. *Striae* **14**, 174–176 (1981).
2. Ehnvall, B. *et al.* Catchment characteristics control boreal mire nutrient regime and vegetation patterns over ~5000 years of landscape development. *Sci. Total Environ.* 165132 (2023)
3. Wang, B. *et al.* Biogeochemical influences on net methylmercury formation proxies along a peatland chronosequence. *Geochim. Cosmochim. Acta* **308**, 188–203 (2021).
4. Ågren, A. M., Hasselquist, E. M., Stendahl, J., Nilsson, M. B. & Paul, S. S. Delineating the distribution of mineral and peat soils at the landscape scale in northern boreal regions. *EGUsphere* 1–23 (2022).
